# Protective hepatocyte signals restrain liver fibrosis in metabolic dysfunction–associated steatohepatitis

**DOI:** 10.1172/JCI179710

**Published:** 2024-04-01

**Authors:** Marcella Steffani, Yana Geng, Utpal B. Pajvani, Robert F. Schwabe

**Affiliations:** 1 Department of Medicine, Columbia University, New York, New York, USA.; 2 Institute of Human Nutrition, New York, New York, USA.; 3 Columbia University Digestive and Liver Disease Research Center, New York, New York, USA.; 4 Herbert Irving Comprehensive Cancer Center, New York, New York, USA.

## Abstract

Metabolic dysfunction–associated steatotic liver disease (MASLD) affects nearly 40% of the global adult population and may progress to metabolic dysfunction–associated steatohepatitis (MASH), and MASH-associated liver fibrosis and cirrhosis. Despite numerous studies unraveling the mechanism of hepatic fibrogenesis, there are still no approved antifibrotic therapies. The development of MASLD and liver fibrosis results from complex cell-cell interactions that often initiate within hepatocytes but remain incompletely understood. In this issue of the *JCI*, Yan and colleagues describe an ATF3/HES1/CEBPA/OPN pathway that links hepatocyte signals to fibrogenic activation of hepatic stellate cells and may provide new perspectives on therapeutic options for MASLD-induced liver fibrosis.

## The cellular crosstalk in metabolic dysfunction-associated liver disease

Chronic liver disease (CLD) kills up to two million people annually, with the majority of patients dying from progression to liver cirrhosis and hepatocellular carcinoma (1). Metabolic dysfunction–associated liver disease (MASLD) affects nearly 40% of the global adult population and is becoming the leading cause of CLD (2). MASLD may lead to the development of metabolic dysfunction-associated steatohepatitis (MASH) and liver fibrosis, the key determinant of outcomes in patients with CLD and MASLD (3, 4). Fibrosis may progress to cirrhosis and liver failure and also predisposes one to the development of hepatocellular carcinoma (4, 5). Fibrosis results from an imbalance between increased extracellular matrix (ECM) production and insufficient ECM degradation (6). While hepatic stellate cells (HSCs) exert a central role in fibrogenesis (7, 8), the initial insult usually occurs in hepatocytes due to metabolic stress, injury, or infection, triggering complex cell-cell interactions that promote the progression of MASLD and the development of liver fibrosis (9). Thus, deciphering hepatocyte signals in the complex intercellular crosstalk that contribute to the activation of HSCs and the development of fibrosis may provide a deeper understanding of the pathophysiology of liver fibrosis. Moreover, with the advent of hepatocyte-targeted siRNA and antisense oligonucleotides, hepatocytes can be therapeutically targeted, whereas targeting of HSCs remains to be clinically established (10–12).

## Hepatocyte CEBPA protects from MASH-driven liver fibrosis

In this issue of the *JCI*, Yan and colleagues reported a reduction of CCAAT/enhancer-binding protein α (CEBPA) in patients with MASH (13). This reduction of CEBPA occurred selectively in hepatocytes and tracked with MASH progression, with a negative correlation between CEBPA mRNA and fibrogenic gene expression. The authors identified two transcriptional repressors, ATF3 and HES1, as candidates responsible for repressing CEBPA, and subsequently demonstrated that their overexpression in primary hepatocytes suppressed CEBPA expression (13). Although it is known that CEBPA has a key role in hepatocyte maturation and liver function, the neonatal lethality of global CEBPA-KO mice has prevented a deeper understanding of its role in adulthood and disease processes including MASH (14, 15). To better understand the role of CEBPA, Yan and authors created mice with constitutive or inducible deletion of CEBPA in hepatocytes. Lack of CEBPA in hepatocytes increased steatosis and development of fibrosis on regular chow and high-fat diets (13). To reproduce the 50% reduction of CEBPA seen in MASH patients, the authors generated mice with heterozygous CEBPA deletion, which also led to increased fibrosis and inflammation in the setting of MASH. Postnatal deletion of CEBPA led to similar results but predominantly affected hepatic fibrosis rather than hepatic lipids. Mechanistically, the authors found that hepatocyte CEBPA actively restrained hepatic fibrosis by negative regulation of the transcription of osteopontin (OPN) (13). OPN, encoded by *SPP1*, is an extracellular cytokine-like protein that activates HSCs and has been shown to be secreted by hepatocytes in MASH (16–18). CEBPA binding to CEBPRE1 directly represses OPN expression. Further, restoring the expression of CEBPA in hepatocytes via AAV8-TBG-*Cebpa* or inhibiting OPN expression by AAV8-mediated silencing ameliorated MASH and fibrosis in mice (13).

## Conclusions and implications

**Figure 1:**
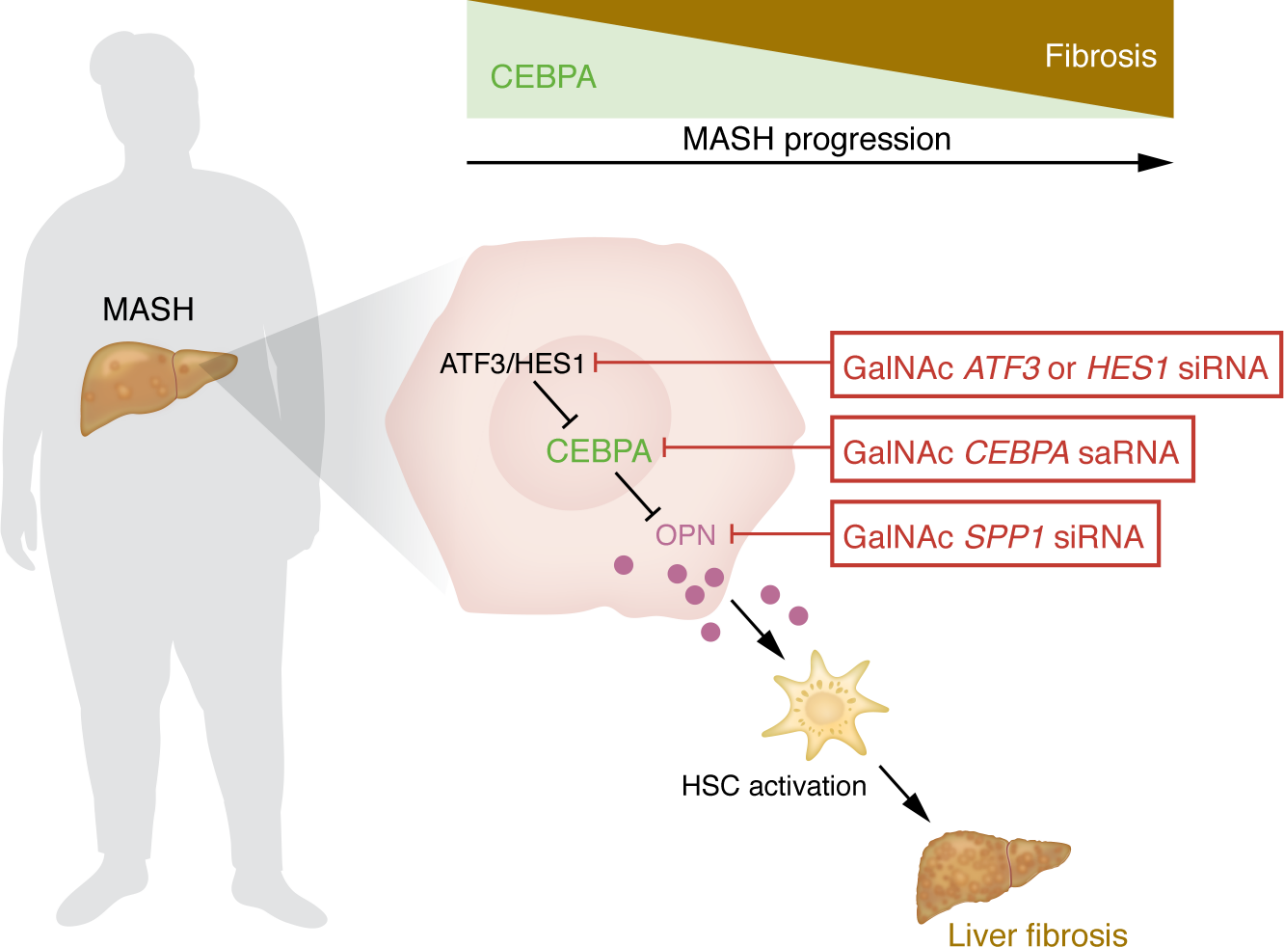
CEBPA confers antifibrotic effects via osteopontin suppression. Activation of ATF3 and HES1 in hepatocytes leads to a progressive suppression of CEBPA that tracks with MASH progression, triggering increased expression and secretion of OPN, encoded by *SPP1*, from hepatocytes. Hepatocyte-derived OPN induces the activation of HSCs and thereby contributes to liver fibrosis. Hepatocyte-specific silencing of *ATF3*, *HES1,* or *SPP1,* or activation of *CEPBA* expression via GalNAc-coupled siRNA or saRNA, respectively, could be used to inhibit fibrogenesis in MASH.

In summary, these studies uncovered a protective signal in hepatocytes that constrains fibrogenesis and is downregulated in patients with MASH (13) (Figure 1). Yan et al. (13) provide a deeper understanding of cellular crosstalk in MASH and, in particular, highlight aberrant hepatocyte signals as a trigger or amplifier of fibrosis progression. Accordingly, hepatocellular CEBPA and its upstream regulators or downstream targets may open up new therapeutic avenues. For example, one clinically feasible and FDA-approved therapeutic option involves the sugar molecule N-acetylgalactosamine (GalNAc), which binds to hepatocytes and may be conjugated to siRNA for hepatocyte-selective silencing or activation (10), e.g., of CEBPA, its upstream activators or downstream targets (Figure 1). However, several open questions must be addressed before considering this CEBPA-dependent, hepatocyte-HSC signaling axis as a potential therapeutic target. It would be important — in addition to causation experiments in mice and altered expression in patients — to determine the effects of CEBPA on outcomes in patients with MAFLD. Furthermore, the decrease of CEBPA during MASH may not occur without reason and may have yet-unknown functions in the regulation of metabolism, regeneration, or other adaptive responses. Hence, a better understanding of the fibrosis-independent effects of CEBPA in the context of MASH will be important. Another critical question involves determining the most suitable targets within this profibrogenic hepatocyte-HSC axis. Upstream regulators such as ATF3 or NOTCH/HES1, CEBPA itself, or downstream targets such as OPN (Figure 1). For example, a wide body of literature has shown amelioration of MASLD by ATF3 inhibition (19, 20); however, mice with global deletion of ATF3 display increased liver injury after ischemia-reperfusion injury (21) and it is currently unknown whether hepatocytes or other cell types impart the ATF3-protective effect. Furthermore, hepatocellular ATF3 likely has broader roles in MASH than the downregulation of CEBPA, which needs to be studied in more detail. Likewise, while the number of HES1^+^ hepatocytes increase in patients with MASH (18), HES1 may have similar adaptive roles. HES1 has been linked to NOTCH activity and glucocorticoid signaling in MASLD (18, 22), but functional studies using hepatocyte-specific deletion or silencing of HES1 are lacking. Studies in hepatocellular carcinoma (HCC) suggest that small activating RNA-based therapy of CEPBPA, which has entered phase I trials (23), may also be considered. Finally, OPN, as a key downstream target of CEBPA and an established mediator of fibrogenesis (13, 16–18), represents another potential target. However, the contribution of hepatocyte-derived OPN, although established in Yan et al. (13) and in previous studies (18), needs to be further confirmed, particularly since OPN from other cellular sources seem to play a role in fibrogenesis and may even exert disease-promoting functions (24). For all interventions, the long-term effects on the development of HCC, which is another main contributor to death in patients with advanced MASH, needs to be studied. Ideally, inhibition of the ATF3/HES1/CEBPA/OPN hepatocyte-HSC axis will inhibit fibrosis and prevent HCC formation in parallel.

## Version 1 04/01/2024

Electronic publication
